# Supplementary material for: Marine ice-cliff instability modeling shows mixed-mode ice-cliff failure and yields calving rate parameterization
Source: Nat Commun. 2021 May 11;12:2701. doi: 10.1038/s41467-021-23070-7 (PMC8113328; doi:10.1038/s41467-021-23070-7)
Supplement: Supplementary file 1 — Supplementary Information [file 41467_2021_23070_MOESM1_ESM.pdf]

Marine ice-cliff instability modeling shows mixed-mode ice-cliff failure and yields calving rate parameterization

Anna J. Crawford, Douglas I. Benn, Joe Todd, Jan A. Åström, J.N. Bassis, Thomas Zwinger

Supplementary Information

Supplementary Note 1: HiDEM

HiDEM, in its standard form, is a brittle-elastic model ($HiDEM_{be}$) that simulates elastic deformation and fracture of ice. Ice is represented by 3D arrays of particles (here, hexagonal close packed lattices) linked by breakable elastic beams. The user defines the particle size and bulk ice density, through which particle mass is derived. A HiDEM simulation is initialised with a percentage of pre-seeded broken bonds, which represents the degree of micro-damage within the ice. All initially formed beams are assigned a width that is proportional to the particle size, fracture strain threshold and a stiffness value that relates to the Young's modulus of ice[1,2].

Stresses are calculated on the inter-particle beams as the ice deforms, with particle displacement being calculated at a time step of $1e^{-4}$ s with a discrete version of Newton's equation of motion. Particle movement is dependent on applied torques and forces (gravity, buoyancy, overburden pressure) and damping factors (viscous drag, bedrock friction, inelastic collisions and oscillations). The inter-particle connections are represented as Euler-Bernoulli beams, which simulate linear elastic deformation under load and is applicable for small strain magnitudes[1,2]. Fracture occurs when the strain imposed by tensile or bending forces on a beam exceeds the fracture strain threshold. The fracture stress threshold can be derived by multiplying the strain threshold with the Young's modulus of ice[1,3]. Un-bonded particles can also interact through inelastic collisions in which a velocity-dependent damping force is applied and through which energy can be dissipated[1].

A further comprehensive description of HiDEM structure is provided in the Supplementary Information of van Dongen et al[1]. We note here that our main simulation series, which underpins the ice-cliff failure retreat rate parameterization development, is conducted with the standard HiDEM structure and parameter settings. As noted in the main text, the HiDEM lattice structure and yield strength combine to represent strong ice that is largely undamaged when initialised with the standard percentage of pre-seeded broken bonds. HiDEM simulations are initalized with a percentage of broken bonds to represent a small degree of micro-fracturing. Fracture is not permitted at the very onset of a HiDEM simulation to avoid the breakage of bonds due to shocks as the system settles.

Supplementary Note 2: Buoyancy-driven calving versus structural ice-cliff failure

We consider buoyancy-driven calving to occur when a glacier terminus reaches a state of hydrostatic disequilibrium without the removal of a sub-arial cliff due to stress-induced failure. The buoyancy-driven calving mechanism plays out through buoyancy forces initiating upward rotation, basal fracturing and iceberg calving via outward block rotation[4]. We observe structural ice-cliff failure in our simulations when full thickness retreat follows the failure of sub-arial ice cliffs as a result of stress concentrations in the vicinity of the waterline. In the viscous deformation to tensile failure mode, stress concentrations result in waterline bulging and a forward lean and fracture ultimately initiates through surface crevassing. Ice-cliff failure via shear localisation also results in the collapse of the sub-arial cliff. In this mode, the slumping of the ice cliff causes the remaining ice column to calve due to its super-buoyant state. Though buoyancy forces are influential in this mode of ice-cliff failure, it is differentiated from the buoyancy-driven calving mechanism by its initiating with the failure of the sub-arial ice cliff due to stresses exceeding the finite strength of ice.

Supplementary Note 3: Further information regarding the results presented in Table 1 and subsequent calving events

We test for structural ice-cliff failure over a viscous deformation period of 50 d, using geometries exported from Elmer/Ice. The lowest cliff height (H_c) at which structural failure initiates is 136 m, this occurring for the normal bed conditions with T_{ice} = -5 °C. Glacier simulations with colder T_{ice} (-10 and -20 °C) do not structurally fail within the 50 d window until H_c reaches 161 m. This is due to the slower rate of viscous deformation at these temperatures. We expect that ice-cliff failure will occur with a time to failure (TtF) > 50 d for these scenarios as long as other calving processes do not occur and modify the strain distribution. The low calving retreat rate ($\hat{C}$) for these cases can be predicted with the MICI calving rate parameterization that this article contributes.

Test simulations are run to showcase scenarios in which viscous deformation affects the timing and mechanism of succeeding calving events (Supplementary Fig. 1). The first test simulation (ice thickness (H) = 1000 m, T_{ice} = -5 °C) shows a switch in calving mechanism due to surface lowering. The viscous deformation leading to the initial failure event lowers the cliff height to 95 m. This lowering of 40 m puts the glacier into a state of buoyancy and the subsequent calving event is driven by the buoyant-calving mechanism and not by ice-cliff failure. The surface slope for this scenario almost doubled due to the long period of viscous deformation leading to the initial calving event. If a glacier remains grounded and surface slope increases it is anticipated that the greater driving stresses and quicker deformation will quicken failure if not countered by mélange back force.

The second test simulation (H = 2000 m, T_{ice} = -20 °C) shows a slowing of calving rate after the first failure event. In this case, surface lowering that occurs prior to the first cliff failure decreases H_c from 339 to 299 m. The TtF of the subsequent calving event is elongated from 1.5 to 9 d and has an associated retreat of 120 m. This equates to a calving rate of 13 m d^{-1}, whereas the calving retreat rate parameterization predicts 48 m d^{-1}.

The last test case (H = 2000 m, T_{ice} = -5 °C) is an example of run-away cliff failure initiating. The initial ice-cliff failure creates a new calving face with H_c = 347, which is 8 m taller than the original H_c. The

subsequent calving event for this case occurs within the first timestep of the Elmer/Ice simulations (TtF = 0.25 d; $\hat{C}$ = 1200 m d^{-1}). Our parameterization predicts a calving rate of 543 m d^{-1}. The temporal resolution of our Elmer/Ice simulations influences the large model error of this rapidly failing ice cliff. Unlike the previously discussed test cases, the structural failure of the H = 2000 m, T_{ice} = -5 °C simulation is not decelerated by surface lowering between calving events, which sets calving faces with such great thicknesses up for catastrophic retreat. For the cases with H = 2000 m, the surface slope minimally changed in the short period of viscous deformation prior to the initial failures.

Supplementary Note 4: Further information regarding the sensitivity testing for the viscous deformation – brittle failure simulation series

These simulations are conducted for well-grounded glaciers with strong, undamaged ice and limited slip through a one-way coupling of Elmer/Ice and the standard, brittle-elastic implementation of the Helsinki Discrete Element Model (HiDEM$_{be}$) as described in the Cliff Failure via Viscous Deformation and Tensile Failure and Methods sections of the main article.

Basal friction: The basal slip values assigned in Elmer/Ice are selected to realise reasonable glacier velocity values for three bed conditions: normal slip (B_n), high slip (B_h) and approaching frozen (B_f) conditions. We provide retreat rate parameterization fits for these three regimes with T_{ice} = -20 ˚C, as well as fits for normal-slip glaciers with T_{ice} = -10 and -5 ˚C. For test simulations with T_{ice} = -20 ˚C, decreasing basal slip by an order of magnitude (B_f) hastens failure by causing a more severe degree of forward lean at the calving face after a given duration of viscous deformation as the outward deformation of the subaerial cliff outpaces slip at the glacier base (Supplementary Fig. 2). This causes the critical lean angle to be reached more quickly and hastens failure. The opposite is found when basal slip is increased by an order of magnitude (B_h). Enhanced basal slip allows the glacier base to advance at a pace closer to that of the subaerial cliff and the critical angle of forward lean is not reached as quickly (Supplementary Fig. 2).

For example, TtF decreases by 17% for H_c = 255 m when bed conditions were modified from B_n to B_f. When bed slip is increased to represent B_h, TtF increases by 43% to account for the greater length of time needed for the critical forward lean angle to be realised. Similar changes to TtF are observed when friction is increased for a thicker glacier scenario (H_c = 424 m); TtF quickens by 50% when slip is decreased by an order of magnitude, and slows by 75% when increased by an order of magnitude. (Table 1).

Due to differences in the evolved calving front geometries, retreat magnitude is smaller (greater) for the higher (lower) friction scenarios. While TtF also always decreases (increases) for conditions with greater (lower) friction, how $\hat{C}$ is modified is dictated by a combination of the changes in both TtF and retreat magnitude.

Degree-of-buoyancy: We do not find that buoyant disequilibrium influences the initial failure of glaciers with $H_c \geq 136$ m. However, HiDEM_{be} particle displacement fields suggest that buoyancy mechanisms could impact the subsequent calving of the thinner glaciers that structurally failed, these being glaciers with starting cliff heights between 136 and 169 m. Our investigations do not consider buoyancy-driven calving, and future work is required to understand the role of buoyancy forces in the evolution of these cases.

In test simulations, adjusting a simulation to floatation impacts the glaciostatic-hydrostatic force balance and, consequently, viscous deformation rates. The degree-of-buoyancy test simulations differ depending on whether H or H_c remained static when the 1500 m thick glacier (H_c = 255 m) simulation is adjusted to buoyancy. TtF is increased by 5 d when the waterline is adjusted to simulate buoyant conditions for a static H = 1500 m. This is a consequence of reduced viscous deformation resulting from increases in hydrostatic pressure associated with the 66 m decrease in H_c. Adjusting H to buoyancy while maintaining a static cliff height (H_c = 255 m) hastens failure by 7 d due to the increasing longitudinal, englacial stresses associated with the thicker glacier. Observed basal fractures generated due to the imposed buoyancy condition do not become pervasive enough to initiate calving and outward block rotation. Failure ultimately still occurs via subaerial cliff failure in these simulations of buoyant glacier termini with limited basal slip.

The bed and surface slope of our simulations is chosen to broadly represent the setting of Thwaites Glacier. We do not test the sensitivity of our simulations to modifications to basal or surface slope. Increasing or decreasing the ice thickness gradient will work to hasten or slow failure[5] and will modify the placement of the fitted curves in Figure 2.

Supplementary Note 5: Further information regarding the conservative aspect of the MICI calving rate parameterization

Buoyancy: As described in the previous section of this document, our Elmer/Ice – $HiDEM_{be}$ simulation series shows that TtF decreases if H is adjusted (increased) to buoyancy while H_c remains static. Therefore, assuming well-grounded conditions for a given H_c will result in a conservative calculation of $\hat{C}$.

Undercutting: Melt-induced undercutting results in a similar terminus geometry as the forward lean caused by viscous deformation of an ice cliff, similarly imposing tensile stresses on the glacier's surface that can lead to surface crevassing[4]. We do not investigate the influence of enhanced melting of the submerged portion of the terminus in sensitivity tests. However, melt undercutting will augment the forward lean angle of the simulated glaciers, thereby hastening TtF and increasing $\hat{C}$.

HiDEM structure initialisation, and shear localisation: The resolution of our HiDEM simulations was limited by computing power. However, increasing resolution (through decreasing particle size) in $HiDEM_{be}$ results in greater fracture prevalence[1]. This is supported by our finding that TtF is 2.1 times greater for a glacier with $H_c = 255$ m when particle size is increased from 20 to 35 m. Our reported TtF results are therefore conservative when considering the impact of particle size, as TtF would decrease if a smaller particle size was assigned in $HiDEM_{be}$. This would then cause an increase in the derived values of $\hat{C}$.

Supplementary Note 6: Failure in standard $HiDEM_{be}$ simulation without prior viscous deformation

Before viscous deformation is considered, basal fractures are observed in $HiDEM_{be}$ but these fractures do not penetrate the ice thickness (Supplementary Fig. 3). This is potentially constrained by the retrograde topography, and the particle displacement field diminishes over the simulation. This highlights that

structural ice-cliff failure does not occur in our HiDEM_{be} simulations of strong, undamaged ice without prior viscous deformation.

Supplementary Note 7: Kinetic energy scaling of collapse through brittle visco-elastic flow

As cliff failure occurs, the potential energy of the glacier terminus is transformed into kinetic energy that is eventually dissipated to the environment. Kinetic energy (E_{kin}) can therefore be used as a measure of the amplitude of cliff failure over varying *H*. A series of HiDEM_{ve} simulations shows E_{kin} increases with ice thickness, which we represent through a scale factor (SCL) that is applied to the sea level and ice surface of the model domain. Estimated bed friction is scaled using a separate friction scale factor (*f*), with low values of f (10^{-4} to 10^{-5}) being used to induce sliding. Kinetic energy will depend on SCL and *f*, as well as the duration of the failure event. In particular, the relative acceleration of the kinetic energy will depend on these parameters. Using HiDEM_{ve}, we test a scaling relation of the form:

$$E_{\text{kin}} = \text{SCL}_e f^{S_e} g(t/(\text{SCL}_t f^{S_t})),, \qquad \text{(S1)}$$

where SCL_e and SCL_t are functions that depend on SCL, S_e and S_t are constants, *t* is time and *g* is a scaling function. HiDEM_{ve} simulations are conducted across a range of thickness (represented by SCL) (Supplementary Fig. 4), with the results collapsing onto a single valued function, *g* in equation (S1), with S_t = -0.09 and S_e = -0.87. This supports our scaling hypothesis in equation (S1) and indicates that E_{kin} increases with a corresponding decrease in bed friction or increase in *H*. There is also a significant increase in SCL_{e} with increasing *H*, indicating collapse of the entire terminus region for the thickest glacier (~SCL = 2.5 or *H* = 2200 m) (Figs. 3e and Supplementary Fig. 4b).

For the elastic-brittle case, simulated through the Elmer/Ice – HiDEM_{be} workflow, it is straightforward process to measure retreat magnitudes and derive retreat rates via distinct calving events like those illustrated in Figure 1**.** In the brittle visco-elastic case, the shear stress near the calving face has risen so

high that the glacier response is no longer purely viscous, but has become a highly complex mixture of viscous deformation and brittle fracture down to the microscopic scale. This means that distinct calving events can no longer be identified, but the terminus region has entered a state of extended collapse like that shown in Fig. 3. This induces an exponentially increasing instability with cliff height (i.e. exponentially increasing kinetic energy defined by equation (S1) and Supplementary Fig. 4). However, as demonstrated by figures 3d and 3e, this type of collapse very rapidly lowers the ice surface and forms a very dense mélange or ice tongue that would quickly restore stability. It is therefore reasonable to view this highly unstable brittle visco-elastic regime as an upper limit to how high an ice cliff can become before a rapid transformation of it quickly restores stability. The rate of retreat will then depend on mélange properties and the rate at which it can be evacuated for the embayment or fjord, as proposed by Schlemm and Levermann[6]. For glaciers that flow into a wide embayment, such as Thwaites Glacier in West Antarctica, the potential for mélange movement to be inhibited by bathymetric highs will be of critical importance for providing the necessary resistance (Table 1) to stabilise a calving face.

Supplementary Figures

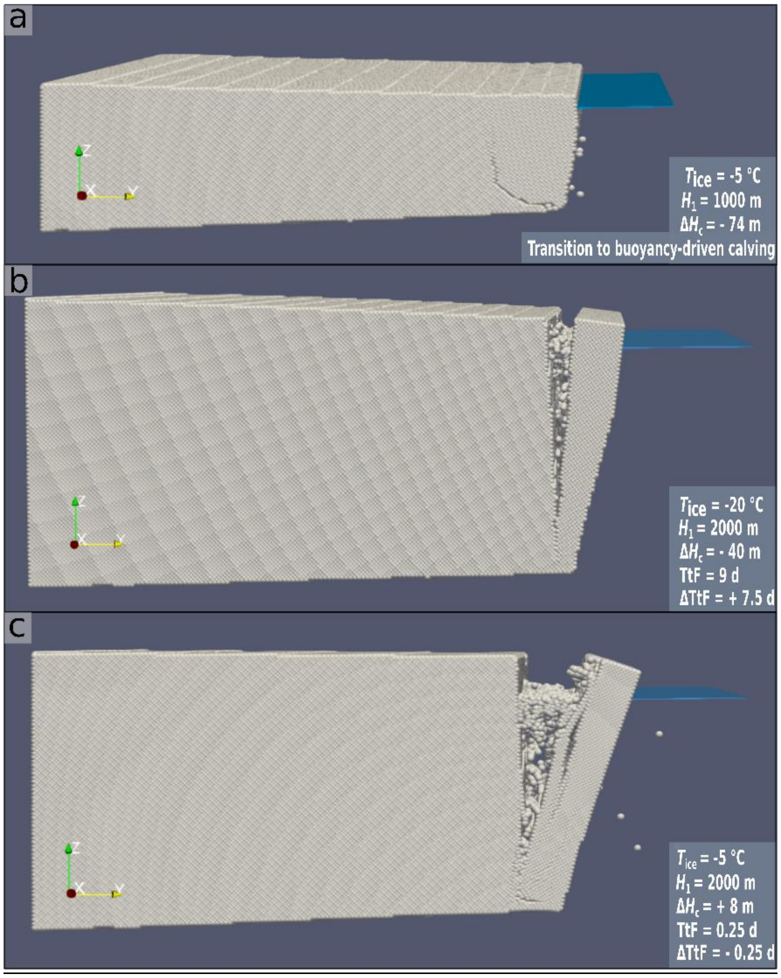

Supplementary Figure 1: Examples of how viscous deformation leads to alteration of mechanism and timing of succeeding calving events in the brittle-elastic version of the Helsinki Discrete Element Model ($HiDEM_{be}$). Thicknesses (H) are those of the calving face prior to deformation and the first calving event. Change in cliff height (ΔH_c) is calculated from cliff heights prior to the viscous deformation leading to the first and second cliff failure events. (a) Transition to buoyancy-driven calving for the thin (H = 1000 m), T_{ice} = -5 ˚C scenario. (b) Time to failure (TtF) slowing for the H = 2000 m, T_{ice} = -20 ˚C scenario due to surface lowering. (c) TtF quickening for the H = 2000 m, T_{ice} = -5 ˚C scenario. Blue planes denote waterlines.

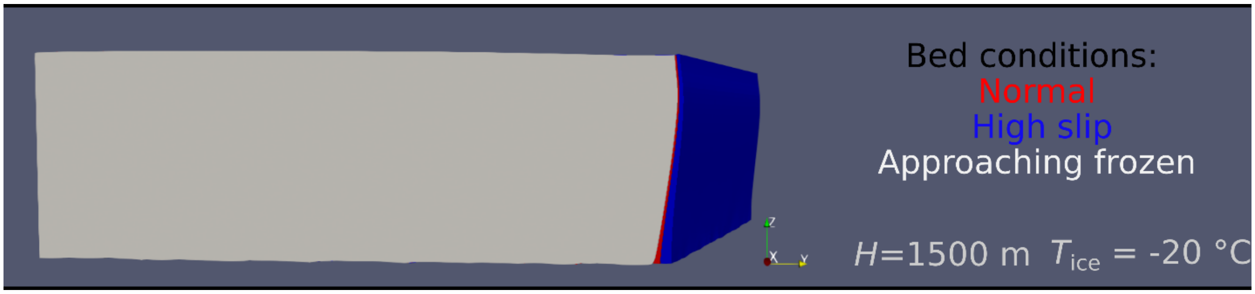

Supplementary Figure 2: Change in lean angle of the calving face after 25 d of viscous deformation in Elmer/Ice for three varying bed conditions. This is a longer duration that necessary to cause failure in the brittle-elastic version of the Helsinki Discrete Element Model ($HiDEM_{be}$). It is used here to better illustrate the differences in deformation. Bed conditions B_n, B_h and B_f form the main text are represented here as normal (red), high basal slip (blue) and approaching frozen (grey), respectively.

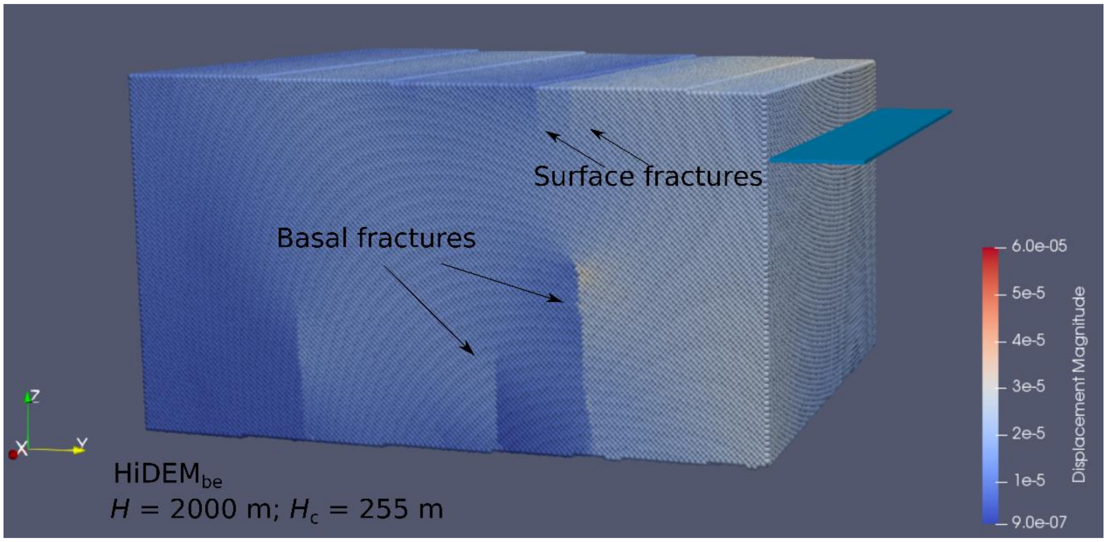

Supplementary Figure 3: A vertical ice cliff in the brittle-elastic version of the Helsinki Discrete Element Model ($HiDEM_{be}$) shows fractures initiate at the base and surface of the 2000 m thick calving face composed of strong, intact ice. Pervasive basal fracturing is constrained by the retrograde topography. H = thickness, H_c = cliff height. The blue plane denotes the waterline.

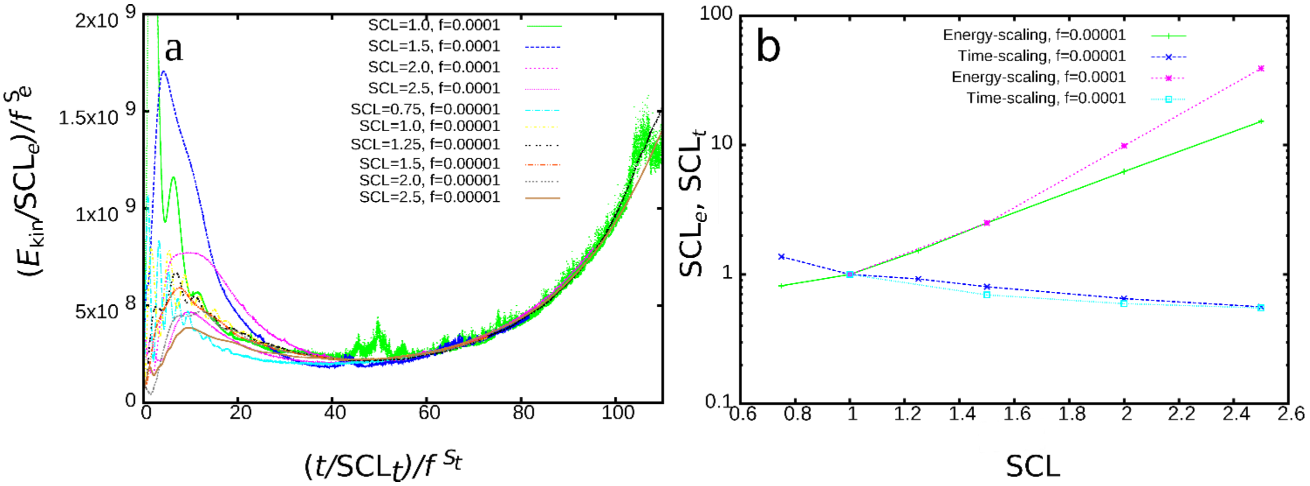

Supplementary Figure 4: Influence of calving front thickness (*H*) on kinetic energy. a) Equation (S1) tested against simulation results for SCL= 0.75 to 2.5, and $f = 10^{-4}$, 10^{-5}. This plot illustrates the collapse of results onto a single function (*g* in equation (S1)), supporting the hypothesis that acceleration of kinetic energy can be described by a single scaling function. The x- and y-axes are rescaled time and kinetic energy, respectively (see Methods for further detail). b) SCL_e (energy-scaling) and SCL_t (time-scaling) as functions of SCL. There are obvious qualitative and quantitative differences between the calving rate parameterization of eq (1) and the brittle visco-elastic kinetic-energy scaling equation (S1).

Supplementary References

1. van Dongen, E. *et al.* Tides modulate crevasse opening prior to a major calving event at Bowdoin Glacier, Northwest Greenland. *J. Glaciol.* **66**, 113–123 (2020).
2. Åström, J. A. *et al.* A particle based simulation model for glacier dynamics. *The Cryosphere* **7**, 1591–1602 (2013).
3. Schulson, E. M. The structure and mechanical behaviour of ice. *JOM* **51**, 21–27 (1999).
4. Benn, D. I. *et al.* Melt-under-cutting and buoyancy-driven calving from tidewater glaciers: new insights from discrete element and continuum model simulations. *J. Glaciol.* **63**, 691–702 (2017).
5. Bassis, J. N. & Ultee, L. A Thin Film Viscoplastic Theory for Calving Glaciers: Toward a Bound on the Calving Rate of Glaciers. *J. Geophys. Res. Earth Surf.* (2019) doi:10.1029/2019JF005160.
6. Schlemm, T. & Levermann, A. A simple parameterisation of mélange buttressing for calving glaciers. *The Cryosphere* 15, 531–545 (2021). doi:10.5194/tc-15-531-2021
