# Supplementary material for: Marine ice-cliff instability modeling shows mixed-mode ice-cliff failure and yields calving rate parameterization
Source: Nat Commun. 2021 May 11;12:2701. doi: 10.1038/s41467-021-23070-7 (PMC8113328; doi:10.1038/s41467-021-23070-7)
Supplement: Supplementary file 4 — Description of additional supplementary files [file 41467_2021_23070_MOESM4_ESM.docx]

Description of additional supplementary information

Title: Supplementary Movie 1

Description: The intermediate elastic-brittle and viscous mode of HiDEM<sub>ve</sub> as applied to a modified domain of Thwaites Glacier with limited basal slip, a thickness of 880 m and a cliff height of 118 m. Shear-banding as well as surface and basal crevassing are observed. Calving ultimately occurs through basal crevasse penetration and backward block rotation, after which a dense melange is formed. The timescale of the animation is model specific and does not imply a particular real-world time frame.
